# A scoping review of knowledge, attitudes, and practices in swine farm biosecurity in North America

**DOI:** 10.3389/fvets.2025.1507704

**Published:** 2025-03-03

**Authors:** Maurine C. Chepkwony, Dennis N. Makau, Colin Yoder, Cesar Corzo, Marie Culhane, Andres Perez, Maria Sol Perez Aguirreburualde, André J. Nault, Michael Mahero

**Affiliations:** ^1^Center for Animal Health and Food Safety, University of Minnesota, Minneapolis, MN, United States; ^2^Department of Biomedical and Diagnostic Sciences, College of Veterinary Medicine, University of Tennessee, Knoxville, TN, United States; ^3^Health Science Libraries, University of Minnesota, Minneapolis, MN, United States; ^4^Department of Veterinary Population Medicine, College of Veterinary Medicine, University of Minnesota, Minneapolis, MN, United States

**Keywords:** swine biosecurity, knowledge – attitude – practice, North American pig farming, biosecurity adoption, critical biosecurity practices, foreign animal disease (FAD), risk assessment

## Abstract

Pork is one of the most popular consumer meat choices globally, second to poultry. In the past two decades, the rising demand in pork, has seen pig farming move toward intensive farming methods, characterized by high pig densities which is a risk for swift spread of disease necessitating proper and strict biosecurity adherence to facilitate disease-free conditions and business continuity. North America is the second largest pig producer globally. We conducted a review of available peer-reviewed original publications to scope for available data on the knowledge, attitudes, perceptions, and practices concerning biosecurity among swine producers in North America from the year 2011 to 2022 using the PRISMA-SCr guidelines. Out of the 323 papers that fit our search criteria, we present insights from the 18 papers that were relevant to our study. We summarize key findings on biosecurity practices and propose critical practices for biosecurity adherence. We also present our findings on the complexities that influence producers’ adoption of biosecurity plans and note variations in biosecurity strictness between states and how these are influenced by farm size and perceived disease risk. In conclusion, this review highlights the need for updated assessments of biosecurity practices, leveraging technology particularly machine learning, for risk assessment, and acknowledges the role that demographics and risk perception play in biosecurity adoption. Ultimately, effective biosecurity measures are imperative for safeguarding North American swine production systems against disease threats especially foreign animal diseases like the African swine fever (ASF), foot and mouth disease (FMD) and classical swine fever.

## 1 Introduction

Global consumption of pork has increased greatly, making it the second most consumed meat, representing >34% of global meat consumption (2022 estimates) (1, 42). Studies reveal a 77% growth in pork demand from 1990 to 2022 (2) and with the growing world population and economic growth, the demand is likely to continue growing (2, 3). Consequently, global pig production has grown by about 140% from 1990 to 2021 (42). The USA, Mexico, and Canada ranking among the top 10 pig-producing countries globally (2). This puts North America as the second largest pig producer globally after Asia. With the ever-increasing threat of foreign animal diseases (FADs), such as African swine fever virus (ASFv), and the continued impact of production-limiting effects of endemic diseases, such as porcine reproductive and respiratory syndrome in North American swine populations, a continuous review and reflection on biosecurity practices and threats is crucial to support optimal production, avert disruption of business and protect the global protein value chain.

Global demand for pork has led to intensification in pig production (23, 41). The high swine densities in farms coupled with increased trade in swine and swine products create an environment conducive to rapid disease transmission. Locally, endemic diseases as well as transboundary diseases are threats to the swine industry and associated commercial trade in addition to human health in some instances (3–5).

Biosecurity is the implementation of measures intended to prevent/reduce the risk of the introduction and spread of disease-causing agents. Biosecurity is guided by 3 principles which include bio-exclusion, bio-management, and bio-containment (6). Bio-exclusion refers to the prevention of disease from entering the farm and spreading, and relies on external biosecurity measures to keep pathogens out. Bio-management and bio-containment rely on internal biosecurity measures to stop pathogens from spreading within the facility. Bio-management relates to measures taken to prevent spread of infectious disease to uninfected animals within the same barn. These include but are not limited to surveillance, all-in, all-out model, rodent control, cleaning and disinfection of equipment and premises, separation and quarantine of sick and symptomatic swine and adjunctive measures like vaccination etc. On the other hand, bio-containment relates to measures that prevent spread of infectious disease to other barns in the same farm and from potentially spreading to other farms (pathogen exit) like shower in- shower out, cleaning and disinfection of shared equipment like skid loaders etc.

Swine production like other livestock industries is threatened by infectious diseases which result in direct losses to production through mortality, loss of productivity, resulting in food insecurity and loss of business continuity through supply chain disruptions, reduced market value and trade restrictions. Biosecurity helps prevent the need for extreme measures needed to control disease spread. While there are recommended biosecurity standards and principles in North America, implementation by farmers is voluntary (7). Some of the biosecurity guidelines are presented in documents such as the secure pork supply (SPS) plan for ensuring continuity of business in case of the introduction of a FAD into the United States. The SPS plan provides a checklist for pork producers and swine veterinarians to use as a guide for implementing biosecurity measures with the ultimate incentive being the guarantee of continuity of business for the industry in the event of an FAD event (8). Other similar guidelines are in place such as the swine industry foreign animal disease preparedness and response plan (FAD-PReP) manual created by the USDA and APHIS (9). Similarly, the Canadian Pork Council has a guidance document for swine biosecurity in its repository (10).

Effective biosecurity is best achieved through the proactive adoption of preventive attitudes and behaviors among all relevant stakeholders, supported by a well-structured biosecurity plan. Preventive attitudes involve anticipating risks and implementing measures to mitigate them before a biosecurity breach occurs, while behaviors include actions such as cleaning and disinfecting equipment, quarantining new animals, and monitoring for disease symptoms (11). A biosecurity plan serves as a framework outlining specific protocols for disease prevention, detection, and containment, tailored to the species, disease prevalence, and management practices (12, 13). Collaboration among stakeholders—farmers, veterinarians, transporters, and policymakers—is critical for ensuring shared responsibility in the implementation of biosecurity measures (14, 15). The success of biosecurity initiatives heavily depends on fostering compliance through behavioral change, as described by the Theory of Planned Behavior, which emphasizes the role of attitudes, perceived behavioral control, and subjective norms in driving actions (16). Challenges such as resistance to change, lack of knowledge, and perceived costs can hinder compliance, but education, training, and demonstrating the economic and health benefits of biosecurity can effectively address these barriers (17, 18).

In this scoping review, we collate information that is available on the knowledge, attitudes, and practices of the swine industry in North America. We assume that published research is representative of how much focus and funding has been applied to the topic within the region. Sources of funding play a role in shaping research focus and will often tend to concentrate efforts on topics involving high-impact biosecurity. Regular assessment of the biosecurity threats and risks for biosecurity breaches is key to sustaining a disease-free status of individual farms and the subsequent continuation of business in the region. To the best of our knowledge, there is limited information on the assessment of biosecurity status in North America that has been publicly published since 2019. The objective of this paper is to provide a review of biosecurity knowledge, attitudes, and practices (KAPs) within the swine industry, the willingness of players to adopt enhanced biosecurity practices, and potential barriers to adoption. Our work also identifies critical biosecurity practices (CBPs) required for the prevention of disease introduction into North American swine farms.

### 1.1 Review questions

The primary question of our review was to identify the current biosecurity practices of swine producers, including the distribution/implementation of these practices amongst swine producers in North America. Secondly, was to ascertain producer attitudes toward the implementation of biosecurity and biosecurity protocols, and lastly to identify key areas for improvement of biosecurity amongst swine producers within North America.

## 2 Methods

### 2.1 Protocol

This scoping review was conducted according to the framework created by Arksey and O’Malley (19). The steps followed include (1) identifying the research question; (2) identifying relevant studies; (3) selecting studies using inclusion and exclusion criteria; (4) charting the data to extract data from each study; and (5) collating, summarizing, and reporting an overview of the results and lastly; reporting follows the guidelines of the Preferred Reporting Items for Systematic reviews and Meta-Analyses extension for Scoping Reviews (PRISMA-SCR) (20). The PRISMA-SCR checklist is attached as Supplementary data 1.

### 2.2 Inclusion and exclusion criteria

We conducted a review of available peer-reviewed publications to scope for available data on biosecurity practices among swine producers in North America from the year 2011 to 2022 using the Covidence platform (21), this was our defined population, concept, and context (PCC) of our research. The papers also needed to be original publications and contain information on farmer practices and attitudes toward biosecurity, the production system involved, as well as information on their profile. A comprehensive list of our inclusion and exclusion criteria is presented in Table 1.

**Table 1:** Inclusion criteria used for selection of papers to be included in the scoping review.

Inclusion criteria	Exclusion criteria
1. Focuses on swine production systems	Non-swine production systems
2. In English	Not English
3. Study on Biosecurity and Disease Management in Swine Production systems	No mention or focus on biosecurity or disease management
4. Published between 2011 and 2022	Published before 2011
5. The study was carried out in North America	The study was carried out outside of North America
6. Contains information on practices, production systems, the profile of producers, attitudes of producers toward biosecurity, or recommendations about biosecurity	Does not have information on practices, production systems, profile of producers, attitudes of producers toward biosecurity, or recommendations about biosecurity
7. Original research papers	Non-research papers (e.g., reviews, letters to the editor, case reports, etc.)

### 2.3 Search and review process

A comprehensive search strategy was developed by an experienced librarian at the University of Minnesota in collaboration with subject experts on the research team to identify all available research on swine biosecurity in the United States. After several test searches, we settled on using PubMed/MEDLINE and center for agriculture and bioscience (CAB) Abstracts (via Ovid) as together these would provide a sufficiently representative sample of the published literature on the topic.

The final search terms used were:

- PubMed: (pigs or swine) and (farmer* or producer) and biosecurity (all fields).
- CAB Abstracts: ((pigs or swine) and (farmer* or producer) and biosecurity).af. where ‘af’ stands for ‘all fields’.

Searches were run on July 12, 2022 and included a publication date limit of 2011 to current (2022), a 10 year study period. Results were imported into Endnote for de-duplication and then transferred to the Covidence platform (21) where they went through filtration of titles and abstracts followed by full-text review and decisions to keep or exclude was based on the inclusion and exclusion criteria described. A total of 6 reviewers were involved in the process with agreement between at least two reviewers being needed for a paper to move to the next step of the review process. Any discrepancies were resolved either by discussion or review by a third reviewer. Charted data was also extracted by at least two reviewers and agreement was discussed and assessed during analysis. For all the studies that fit our criteria of selection, we looked at what their research aims were, the type of swine production systems they studied, what study design they used to answer their questions, and outlined the internal and external biosecurity practices flagged in their study. The search strategy is outlined in Supplementary data 2.

### 2.4 Content analysis

Data on CBPs as well as risk factors for disease introduction into swine farms was tabulated and tallied by frequency of mention of either exact factor or synonyms of factor, e.g., truck/vehicle/trailer were considered to refer to the same mode of transportation. In the line-by-line analysis, we teased out what the papers were saying about the highlighted factors; this data can be found in Supplementary Table 1.

## 3 Results

### 3.1 Search results and study characteristics

**Figure 1:**
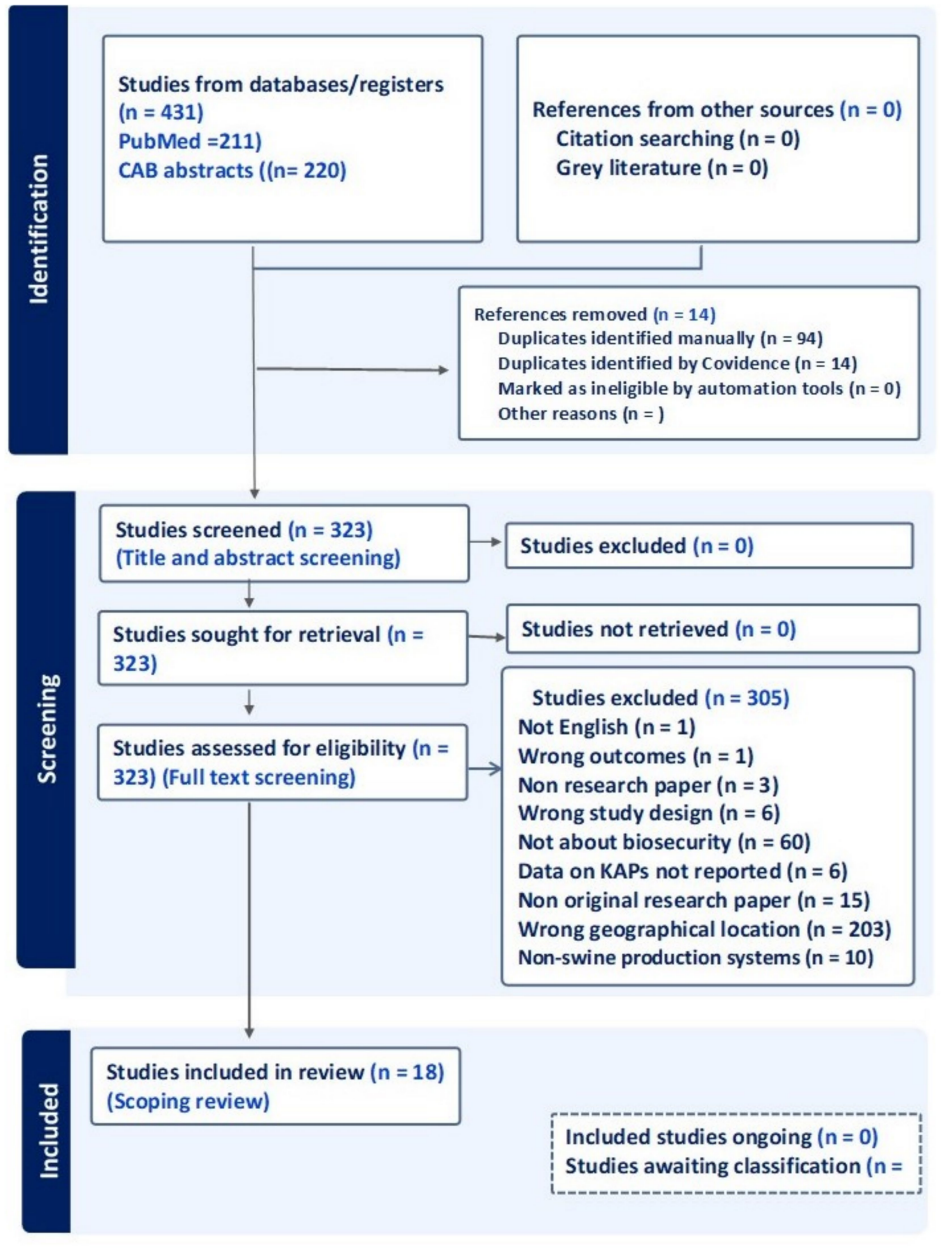
PRISMA flow chart of the paper selection process showing the number of papers in each step and the representation of reasons why excluded papers were left out leading to the final 18 selected.

In PubMed, our search strings produced 211 results and CAB Abstracts produced 220 results, respectively. After deduplication in Endnote, 337 records remained. Covidence was able to remove an additional 14 records, leaving a total of 323 papers. These went through a filtration and review process yielding a total of 18 studies relevant to our review with most of the papers being excluded for being outside our geographical scope (i.e., conducted outside N. America) *n* = 203. Other reasons for exclusion were non-original papers (*n* = 15), data on KAPs not reported (*n* = 6), not about biosecurity (*n* = 60), non-research papers (*n* = 3), and not in English (*n* = 1), wrong study design (*n* = 6). The PRISMA summarizing this is in Figure 1.

### 3.2 Distribution of studies

**Figure 2:**
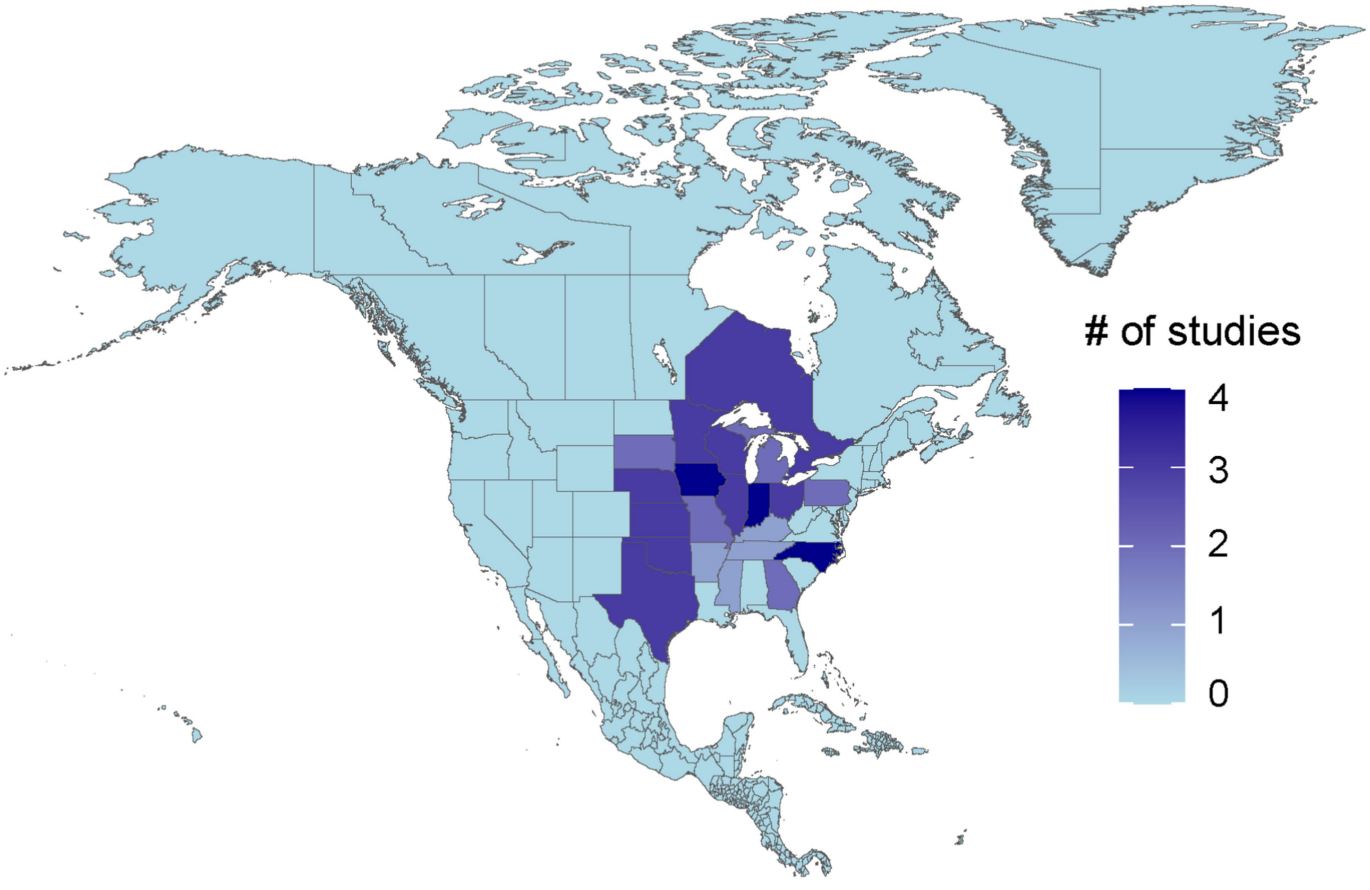
A map highlighting the North American states covered in the research studies included in this scoping review on swine biosecurity conducted between 2011 and 2022. Blue represents states and provinces not represented in the research while purple are the states covered by the research. The deeper the shade of purple, the higher the number of papers that conducted research in that state/province.

While North America comprises Canada, the USA, Mexico, Central America, and the Caribbean, the majority of the included studies originated from the United States (*n* = 15). Canada and the USA are top global swine producers, Mexico’s role is growing with increasing export to neighboring countries and the Caribbean and Central America keep swine largely for domestic market consumption. There were a total of three studies (16.7%) conducted in Canada and no relevant papers were found from Mexico and the other regions. While inclusion of only English-language papers may have excluded Spanish and French studies from the region, a limitation stemming from the team’s constraints in resources, funding, and translation expertise, we attempted to address this limitation by utilizing multilingual databases such as PubMed and CAB Abstracts. Figure 2 maps the geographic distribution of research on swine biosecurity across North America.

**Figure 3:**
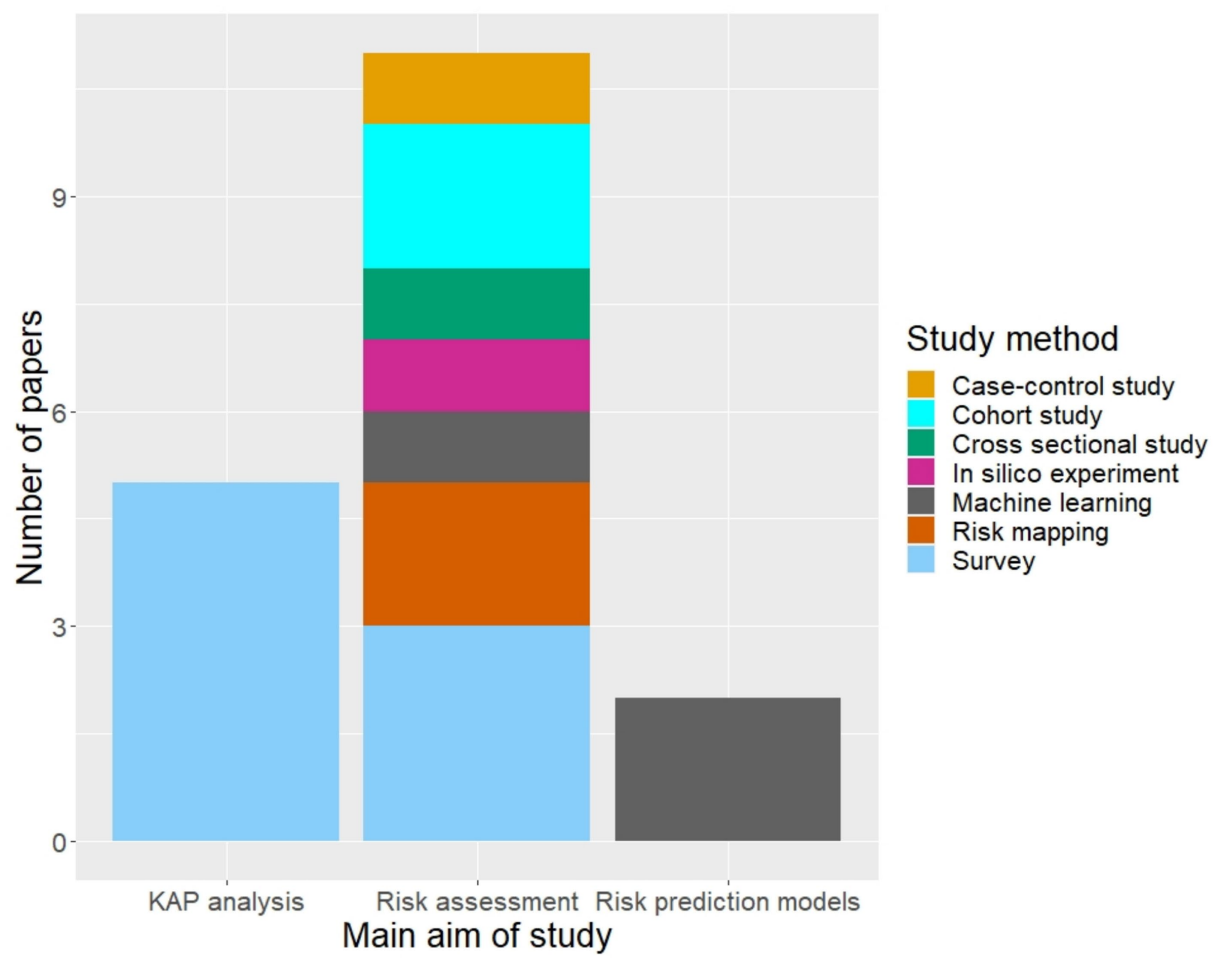
Summary of the types of research published on swine farm biosecurity in North America between 2011 and 2022.

The 18 papers included in this review primarily covered KAP analyses (*n* = 6) and disease-specific biosecurity risk assessments (*n* = 9), such as risk mapping and machine learning simulations to predict swine farm vulnerability to disease (Figure 3). These studies were largely observational, including surveys, cross-sectional, cohort, and case–control studies, with some employing computer-based experiments, such as in silico and machine learning models.

### 3.3 Knowledge

Knowledge about biosecurity practices varied across studies, with 61% (11/18) of the research focusing on mixed systems of swine farming (e.g., small or medium-scale indoor, medium-scale outdoor, indoor or outdoor extensive). However, few studies emphasized greater knowledge gaps about biosecurity and its implementation among backyard or hobby or small scale farmers. Medium-sized farms were defined as having an inventory of 750–2,499 swine each weighing 55 pounds or more, or 3,000–9,999 swine each weighing less than 55 pounds. Farms with inventories smaller than this were classified as small farms, while those with larger inventories were considered large farms (22).

The swine production industry in North America predominantly consists of commercial swine raised in strict indoor confinement systems (23). Despite this, knowledge gaps remain regarding critical aspects of biosecurity, particularly in areas like small-scale or backyard farming, where there is less access to structured biosecurity protocols.

### 3.4 Attitudes and perceptions

Producer attitudes and perceptions significantly influence the adoption of biosecurity measures. The most important factors that influenced a swine producer’s decision to either take up a biosecurity practice or not, were: producers’ perceptions of the risk of disease; personal experience with a disease outbreak on their farm or the neighbor’s farm(s); and effectiveness of a biosecurity practice in reducing the perceived risk. A study by Wu et al. (24) reported that 65.8% of swine producers in the United States of America were willing to adopt biosecurity practices to reduce disease risk to their farms and their neighbors’ farms; however, this percentage reduced to 56% after considering the high cost of implementation. Experience is indeed the best teacher; (24) also found that experiencing an FAD with significant impact on animal health, trade and food security swine disease either personally or on the neighboring farms was the biggest incentive for swine producers to adopt biosecurity protocols.

Additionally, government incentives or payouts partially encouraged adoption of biosecurity practices. Availability of more educational resources on a topic was the least effective in encouraging national adoption of biosecurity practices among swine producers suggesting that while educational materials are important in farmer education, merely increasing the quantity of resources without considering the alignment of these resources with practical needs of farmers may hinder the effectiveness of the tools.

The role backyard farming may play in disease introduction, spread and establishment is often understated and underestimated. Nicholson et al. (23) observed that backyard swine farmers in Pennsylvania had varying perceptions of biosecurity. While they were relatively knowledgeable about highly infectious diseases like swine influenza due to recent education campaigns, they lacked awareness of zoonotic diseases or those with public health implications. However, despite the mentioned campaign, biosecurity practices among backyard farmers in the state were very lax, with the majority having no biosecurity protocols or requirements for visitor access to their animals (23). This highlights the importance of targeted education campaigns tailored to specific producer demographics and risks. Consideration for inclusion of small farmers in education campaigns etc., may potentially benefit the entire industry.

The strictness of biosecurity implementation by swine producers varies by several factors including geographical location, density of swine farms and the associated perceived feasibility of implementation. While biosecurity measures have been implemented to varying degrees, farmers’ attitudes are largely unknown with regards to how they would prioritize adopting these measures and how this impacts the goal to have continuity of business in the event of introduction of a foreign pig disease into the region. The risk of certain FADs getting established in the region is also relatively higher in some states that others. Taking ASFv as an example; Wormington et al. (25) describe California, Oregon, Nevada, Arizona, New Mexico, Oklahoma, Texas, and Florida as having potential risk of ASFV spillover from the sylvatic cycle due to the co-occurrence of the tick vector, feral swine, and domestic swine. While direct swine-to-swine transmission can also occur in these states, the authors list North Carolina and Oklahoma as being of particularly high concern for direct swine-to-swine virus transmission particularly due to high swine farm densities.

### 3.5 Practices

Practices associated with biosecurity measures were evaluated based on their frequency of mention and criticality. The most frequently mentioned practices were vehicle movement management (27.8%; 5/18) and personnel management (16.7%; 3/18) (26). Other practices included the sourcing of animals and semen, manure management, feed handling, pest and wildlife control, and air and water filtration, each cited in 11.1% (2/18) of the reviewed papers (Table 2).

**Table 2:** Summary of the different swine farm biosecurity practices highlighted in the reviewed publications.

Biosecurity practice	Principle	Specific practices
Vehicle movement	Bio exclusion	Cleaning and disinfection of trucks in manned designated areas. Restriction of different vehicles to specified zones, e.g., visitor vehicles park outside PBA.
Disposal of dead animals	Bio-management and bio-containment	Method of disposal. Directionality of removal of dead animals from barn. Location of rendering bin in respect to barns and access to rendering truck.
Manure Management	Bio-management and bio-containment	Frequency of manure removal Location of lagoons in respect to swine housing. Directionality of removal from housing.
Management of feed	Bio-exclusion	Audit and verify feed suppliers. Feed holding time. Clean and disinfect feed trucks. Cleaning feed spills
Perimeter buffer area	Bio-exclusion	Clearly demarcated PBA with specified entry points. Cleaning and disinfection of personnel and trucks at PBA entry points.
Restriction of visitors and staff movement	Bio-exclusion and bio-containment	Presence of specified, monitored entry points. Cleaning and disinfection of personnel and PPE at entry points. Clear defined and marked LOS.
Sourcing of animals and genetic material	Bio-exclusion	
Line of separation (LOS) and shower-in facility	Bio-exclusion and bio-management	LOS and shower-in facility specified at entry to each animal barn and entry to farm where feasible.
Filtration of air and water in swine housing	Bio-exclusion and bio-containment	Filter air coming into and going out of the barn.
Animal–animal contact/separation	Bio-management	
Restriction of movement within the facility	Bio-management and bio-containment	Restriction of staff movement between animal housing. Directionality of flow of staff, animals and equipment, i.e., designated entry and exit of opposite sides.
Presence of defined clean and dirty areas with a defined LOS	Bio-exclusion and bio-containment	Defined LOS for personnel, animals and equipment.
Wearing of personal protective equipment (PPE)	Bio-exclusion, bio-management and bio-containment	PPE should be changed before entry and before exiting each housing/facility. Cleaning and disinfection or disposal of PPE between each animal housing.
Disinfection of pig housing	Bio-management	Should be done between each swine group after all in- all out occupancy. Disinfection of floors during cleaning.

**Figure 4:**
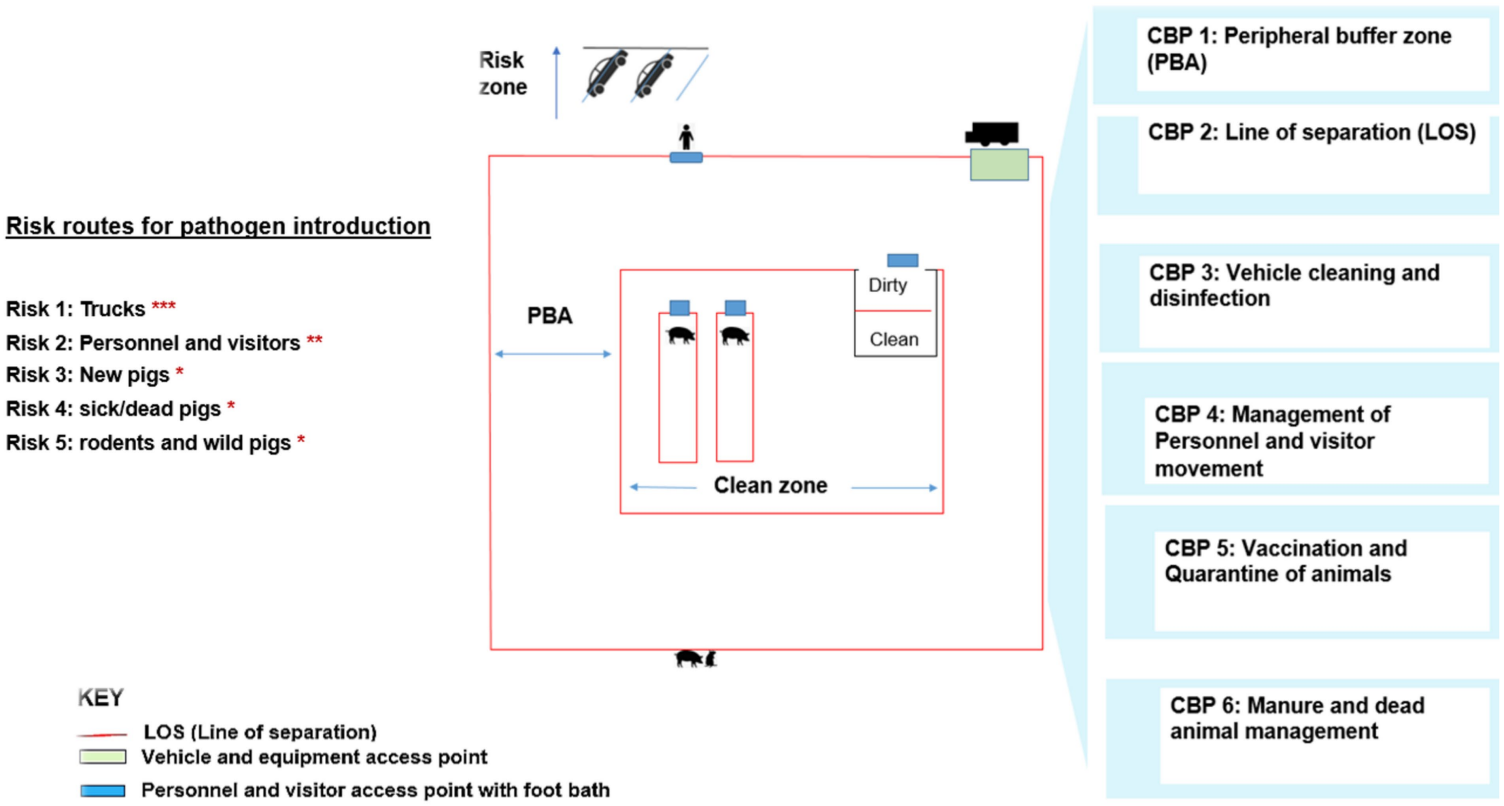
Pictorial of the recommended biosecurity-related checkpoints on a farm, the risks routes of pathogen introduction, and associated critical biosecurity practices (CBPs). PBA is ideally between the perimeter fence on the outer and fencing for the clean zone. Asterisks reflect the ranking of risks based on their frequency of mention in a qualitative scoping review, with *** indicating the highest frequency, ** the second highest, and * moderate frequency.

The reviewed literature emphasized specific biosecurity practices that are critical and warrant attention to mitigate pathogen transmission risks. These included controlling vehicle and personnel movement, managing the removal of dead animals, implementing defined, clearly marked and with specified protocols for crossing the designated access points to biosecurity zones such as the Line of Separation (LOS) and the Peripheral Buffer Area (PBA), and ensuring the proper handling of feed and manure. Biosecurity practices discussed included vehicle disinfection, PPE protocols, and quarantine facilities to mitigate risks associated with pathogen introduction and spread. Vehicle disinfection, PPE protocols, and quarantine facilities to mitigate risks associated with pathogen introduction and spread. We used the frequency if the mentioned practices in our reviewed papers as metrics to identify practices and points in a swine farm’s biosecurity infrastructure and protocols that are crucial for prevention or mitigation of pathogen transmission risks. We refer to these as critical biosecurity practices (CBPs) of biosecurity in swine farms (Figure 4) and borrow our recommendations from the SPS protocol.

We outline here the specific critical biosecurity practices (CBPs) identified through this review:

1. Defined Peripheral Buffer Areas (PBAs): A well-demarcated PBA restricts unauthorized access and minimizes contact between farm operations and external risk factors like vehicles and wildlife.
2. Line of Separation (LOS): Clearly defined zones that separate clean and dirty areas. These are monitored and include biosecurity guidelines for crossing.
3. Vehicle Cleaning and Disinfection: Vehicles used for transport of swine, feed, or equipment should be disinfected at the farm entry point. Shared vehicles between farms present a significant risk of pathogen transmission (26).
4. Personnel and Visitor Movement: Risks of disease introduction by personnel and visitors can be mitigated by footbaths, controlled access points, and mandatory use of personal protective equipment (PPE). A shower-in, shower-out system with designated clean and dirty areas further enhances biosecurity (26, 27).
5. Vaccination and Quarantine: New animals should undergo vaccination and quarantine to reduce the risk of disease introduction. Testing incoming animals for pathogens is also recommended to complement vaccination, especially for diseases without effective vaccines.
6. Manure and Dead Animal Management: Proper handling and disposal practices minimize disease spread within and outside the farm. Disinfection of rendering trucks and placement of rendering boxes outside the PBA are essential measures (26).

The reviewed studies underscored the importance of combining multiple CBPs to achieve effective biosecurity. For instance, LOS and PBAs provide physical and procedural barriers, while practices like vaccination, testing, and personnel management reduce the likelihood of pathogen introduction and spread.

The studies also identified aspects that act as barriers to implementation of biosecurity practices in swine industry in North America. One significant challenge is ensuring active engagement from swine producers in adopting biosecurity measures and implementing risk assessments. Without their full cooperation and trust, even advanced technological solutions may have limited impact. Another barrier is access to comprehensive and reliable data. In vertically integrated or large-scale production systems, much of the necessary data remains proprietary or inconsistently recorded, hindering its use in comprehensive risk analyses.

## 4 Discussion

Here we discuss the salient points that emerge from the 18 relevant papers included in in our review on knowledge, attitudes, perceptions, and practices of swine farmers in North America. We summarized both, macro and micro-trends in biosecurity.

The application of machine learning and data science as risk assessment and prediction tools remains an area in need of research. Machine learning is being used in the region to predict the exposure of individual farms to disease in the event of an outbreak (26). It would be beneficial to the industry’s producers and veterinarians to have the prediction of the vulnerability of farms in the region to diseases like ASF, which have crippled entire production systems, should it spread to regions where it is not endemic (28, 29). The machine learning models can benchmark and measure which biosecurity practices and farm demographic factors are contributing to the risk of exposure, thus identifying weak points within a farm’s biosecurity plan for disease introduction. The use of such technology has the advantage that it can be run at intervals to give a longitudinal assessment of farm vulnerability, essentially aiding the management of virus outbreaks over time (26, 30). With the ever-increasing accessibility to technology and technological advances that can support better disease prevention and risk mitigation, research in the application of different technologies in swine biosecurity management and their role in improving compliance with biosecurity standards and principles in current swine production systems would be beneficial.

While machine learning models provide a promising tool to identify gaps in biosecurity and monitor farm vulnerabilities over time (26, 30), several practical challenges must be considered. One significant challenge is ensuring active engagement from swine producers in adopting biosecurity measures and implementing risk assessments. Without their full cooperation and trust, even advanced technological solutions may have limited impact. Therefore, fostering an understanding of the value these tools bring to farm management is essential for their success (24).

Additionally, access to comprehensive and reliable data presents another significant hurdle. In vertically integrated or large-scale production systems, much of the necessary data remains proprietary or inconsistently recorded, hindering its use in comprehensive risk analyses. Key information, such as farm demographics, movement patterns, and compliance behaviors, may not always be readily available, making it difficult to fully leverage machine learning capabilities. Collaborative efforts to establish data-sharing frameworks and standardized biosecurity metrics are critical to address this gap and ensure these technologies can be effectively applied in practice.

It is evident that the strictness of biosecurity implementation by swine producers varies by several factors including geographical location, density of swine farms and the associated perceived feasibility of implementation. However, factors like swine density could have mixed effects on implementation of biosecurity practices. For instance, farms in higher swine farm density areas in Ontario, Canada were more likely to adopt higher biosecurity practices; the reverse was reported in Iowa, USA (40). The high pig density in Iowa was found to be associated with decreased rates of on farm biosecurity adoption an observation that (31) refer to as the ‘Iowa variable’ in their paper. This has been linked to producer perception that disease exclusion using biosecurity measures was not realistically achievable in the high farm density and integrated system of production in Iowa (31). Despite this difference, both studies (31, 40) agreed that larger farms implemented tighter biosecurity, suggesting that the scale of losses a farmer risks is an incentive for the adoption of preventive biosecurity practices.

The role of swine density also extends beyond an individual farm’s biosecurity practices to its broader impact on the industry. High-density farms, although capable of tighter biosecurity, carry a greater risk of amplifying disease spread if biosecurity measures fail. A single outbreak in a high-density farm has the potential to result in substantial financial losses for the producer and disrupt the industry by serving as a disease reservoir, exposing smaller neighboring farms to heightened risks (26). Conversely, smaller farms, while posing a lower risk for catastrophic industry-wide impacts, may lack the resources to implement comprehensive biosecurity measures, making them vulnerable to initial introductions of pathogens.

As exemplified here, adherence to biosecurity standards and principles is differential and this variability could be influenced by, among other factors, inventory and farm size, and perceived risk for disease based on farm location. Essentially there is a gradient of biosecurity practices even among large-scale producers (who may or may not be in the same integrated system) underscoring the need for regular assessment of behaviors associated with risks and how these should be factored in when addressing inter and intra-system transactions. Simply, the traditional one-size-fits-all approach may no longer be tenable and a consideration of biosecurity guidelines within which production systems can operate with some flexibility to refine the biosecurity may be more efficacious and sustainable. Similarly farmer education on existing, new and potential risks and the recommended biosecurity practices to mitigate their impact is important and critical but needs to be adapted to specific farmer demographics, operational needs and particular risks (24).

Additionally, given that the North American swine production landscape is punctuated by a mix of backyard and medium to large commercial production systems, the studies showed that farmers care about both their risk and the risk of their neighbors and could be considered reason enough to implement inclusive biosecurity awareness initiatives and develop guidelines applicable to the different production types.

However, farm attributes notwithstanding, the risk of spread of disease and losses from a disease varies with each infection, depending on the transmission modalities that put a farm at risk, the virulence of a pathogen, and herd susceptibility (based on breed, age, etc.) among other factors. Although the swine industry in the USA has been consolidating over time with 86% of the country’s inventory coming from farms with more than 2000 animals (32), a study by White et al. (33) showed that risk of a farm’s exposure to a disease like Influenza A was the same for all farms having above 200 animals. This means that at least for this pathogen, biosecurity measures to prevent its introduction and spread are necessary regardless of farm size or inventory. Moreover, we underscore the importance of including small and mid-sized swine farms in the risk assessment of FADs and important endemic diseases.

From the published literature, the most important external biosecurity practices discussed were the use of trucks (26, 27, 30, 34, 40) and the sourcing of replacement animals or genetic material (26, 27, 30, 33, 40). The risk is usually higher with trucks shared by several farms, a common and economical practice, thus necessitating good on-farm disinfection protocols and/or PBAs with clear LOS as well as quarantine protocols for any incoming animals and products (26, 27, 30, 34, 40). Farms that share trucks had higher chances of exposure to disease (26). This practice can be influenced by knowledge gaps regarding the role of shared vehicles in disease transmission, as highlighted by Silva et al. (30). Without adequate understanding of how pathogens can persist on fomites like trucks, farm operators may underestimate the risks associated with shared transportation.

It was also hypothesized that farms with many employees were associated with lower incidence of PRRS due to increased attention to personnel biosecurity protocols (26). This observation suggests that attitudes toward risk management may play a role; farms with larger workforces might have stronger incentives to invest in biosecurity measures to safeguard their operations and maintain continuity of business. However, the perception of biosecurity feasibility might differ based on farm size, as large farms tend to have operational connections to multiple sites, which increases the likelihood of pathogen transmission despite strict biosecurity protocols (26, 27). Furthermore, the higher likelihood of exposure to PRRS in large pig inventories (26) underscores the need for knowledge-driven interventions to mitigate risks in these high-density settings. For instance, tailored biosecurity education targeting large-scale producers may help align biosecurity practices with the unique challenges posed by operational complexity and interconnectivity across sites (26).

For internal biosecurity practices to manage and prevent the spread of disease within a farm, restriction of movement combined with clear and enforced biosecurity protocols for visitors and staff at the LOS were the measures most described in the published papers (23, 31, 35, 36). Pudenz et al. (31) in their assessment of the adoption of the SPS plan on biosecurity by swine producers in the USA, discussed the complexities involved in a producers decision to adopt or not to adopt a biosecurity plan. They found that producers who had site specific biosecurity plans have higher adoption of PBA and LOS as described in the SPS plan, compared to farmers who did not have defined site specific biosecurity plans. Overall, a producer’s perception of the feasibility of implementation, risk attitudes, and demographics played a significant role in adoption of biosecurity practices.

Practices like shower-in shower-out, monitoring of movement patterns between animal rooms, and well-maintained foot baths were described with varying levels of implementation on swine farms. Thirdly, manure and dead animal management were important factors in both bio-management and bio-containment practices to minimize the risk of disease spreading from infected farms. We highlight these practices as possible focus areas in future studies, especially since they are not novel practices or concepts to most farmers. While these biosecurity measures have been implemented to varying degrees, farmers’ attitudes are largely unknown with regards to how they would prioritize adopting these measures and how this impacts the goal to have continuity of business in the event of introduction of a foreign pig disease into the region.

The risk of establishment of FADs like the ASF in America is higher in the southwestern states as well as California and Florida due to the presence of both the vector of interest and feral swine that could potentially complete the sylvatic cycle (25). Primarily, the risk of exposure and spread of FADs in the swine industry is defined by the factors influencing the transmission and spread of a particular disease. For the case of ASF, there is concern that increases in temperature and humidity that comes with global warming may influence the spread of tick vectors to new territories where they previously could not survive (37–39). This coupled with reports of feral swine in Canada and some USA states poses a real risk for introduction and successful establishment of ASF in the colder high swine producing mid-western states where swine density is highest. Although at the time of Wormington’s study the risk was low, there is need for biosecurity protocols and regulations that would potentially limit the spread of the disease vectors to the high-producing region of the Midwest; and mitigate the risk for swine-to-swine transmission among swine herds as posited for North Carolina (25).

## 5 Conclusion

No single biosecurity practice is sufficient on its own; a functional biosecurity system relies on the integration of multiple practices. For instance, quarantine measures must be supported by clearly defined Lines of Separation (LOS), proper employee directionality to minimize cross-contamination, and protocols such as shower-in/shower-out and change of clothing between newly quarantined and resident swine. This underscores the importance of a comprehensive, multi-layered approach to biosecurity.

Key findings from this review highlight critical gaps and opportunities for enhancing biosecurity in North American swine farms. These include a scarcity of recent data on biosecurity practices and farmer attitudes, emphasizing the need for updated assessments, particularly given the persistent threat of transboundary swine diseases. Producers’ perceptions of risk and the feasibility of biosecurity measures play a pivotal role in determining the adoption of practices, which suggests that improved outreach and education could drive greater compliance.

By prioritizing critical biosecurity practices (CBPs) such as access controls at the Perimeter Buffer Area (PBA) and LOS, farms can strengthen their disease prevention and containment efforts. However, adoption of these practices remains voluntary, with varying levels of implementation driven by farmers’ perceived incentives and farm-specific demographics. Emerging technologies, such as machine learning, offer exciting possibilities for identifying vulnerabilities and optimizing biosecurity strategies.

Ultimately, the synthesis of existing research provided in this paper serves as a foundation for future studies, while offering actionable insights for swine producers, policymakers, and researchers. Investing in up-to-date data collection, targeted interventions, and tailored education programs can bolster biosecurity measures across the industry. A collaborative and proactive approach is essential to safeguard the health of swine populations, protect livelihoods, and ensure the sustainability of North American swine production systems.

## Data Availability

The original contributions presented in the study are included in the article/Supplementary material, further inquiries can be directed to the corresponding author.
