# Supplementary material for: A scoping review of knowledge, attitudes, and practices in swine farm biosecurity in North America
Source: Front Vet Sci. 2025 Mar 3;12:1507704. doi: 10.3389/fvets.2025.1507704 (PMC11911468; doi:10.3389/fvets.2025.1507704)
Supplement: Supplementary file 2 [file Data_Sheet_1.pdf]

Preferred Reporting Items for Systematic reviews and Meta-Analyses extension for Scoping Reviews (PRISMA-ScR) Checklist

SECTION	ITEM	PRISMA-ScR CHECKLIST ITEM	REPORTED ON PAGE #
**TITLE**			
Title	1	Identify the report as a scoping review.	A scoping review of knowledge, attitudes, and practices on biosecurity in swine production farms in North America.
**ABSTRACT**			
Structured summary	2	Provide a structured summary that includes (as applicable): background, objectives, eligibility criteria, sources of evidence, charting methods, results, and conclusions that relate to the review questions and objectives.	The abstract provides a clear overview of the purpose of the study, methodology, and key findings. It summarizes the scoping review conducted on biosecurity in North American swine farms, highlighting the search methods, inclusion criteria, and the final selection of 18 papers. It mentions the key findings, including biosecurity practices, challenges in implementation, and the need for updated assessments.
**INTRODUCTION**			
Rationale	3	Describe the rationale for the review in the context of what is already known. Explain why the review questions/objectives lend themselves to a scoping review approach.	This scoping review was conducted to address the rising demand for pork production and the associated risk of disease outbreaks in swine farming, particularly in North America. With high-density farming and increasing trade in swine products, the threat of foreign animal diseases (FADs) like ASF and endemic diseases such as PRRS necessitates a review of existing biosecurity knowledge, attitudes, and practices to safeguard the industry.
Objectives	4	Provide an explicit statement of the questions and objectives being addressed with reference to their key elements (e.g., population or participants, concepts, and context) or other relevant key elements used to conceptualize the review questions and/or objectives.	The review aimed to:<br>- Identify current biosecurity practices among swine producers in North America.<br>- Understand producer attitudes toward biosecurity adoption.<br>- Highlight knowledge gaps and propose critical biosecurity practices to improve disease prevention and management.

SECTION	ITEM	PRISMA-ScR CHECKLIST ITEM	REPORTED ON PAGE #
**METHODS**			
Protocol and registration	5	Indicate whether a review protocol exists; state if and where it can be accessed (e.g., a Web address); and if available, provide registration information, including the registration number.	We did not publish a protocol for this review believing that the detailed methods section of this paper would allow replication of our study.<br>The study followed the Arksey and O'Malley framework (2005) and adhered to PRISMA-SCR guidelines. While the protocol was not published, the methodology is detailed in the methods section, outlining inclusion/exclusion criteria and the systematic process for study selection and analysis.
Eligibility criteria	6	Specify characteristics of the sources of evidence used as eligibility criteria (e.g., years considered, language, and publication status), and provide a rationale.	Inclusion criteria:<br>- Focus on swine production systems.<br>- Written in English.<br>- Published between 2011 and 2022.<br>- Conducted in North America.<br>- Original research addressing biosecurity practices, farmer attitudes, and disease management.<br>Exclusion criteria:<br>- Non-swine production studies.<br>- Papers not in English.<br>- Studies conducted outside North America.<br>- Non-research papers (e.g., reviews, editorials).
Information sources*	7	Describe all information sources in the search (e.g., databases with dates of coverage and contact with authors to identify additional sources), as well as the date the most recent search was executed.	Two databases were used for the literature search: PubMed/MEDLINE and CAB Abstracts (via Ovid). Search strategies were developed in collaboration with an experienced librarian and subject matter experts.
Search	8	Present the full electronic search strategy for at least 1 database, including any limits used, such that it could be repeated.	The search strategy included terms such as "pigs," "swine," "farmers," "producers," and "biosecurity." Boolean operators and field-specific filters were applied to ensure a comprehensive search. Searches were conducted on July 12, 2022, and limited to publications from 2011 onward.

SECTION	ITEM	PRISMA-ScR CHECKLIST ITEM	REPORTED ON PAGE #
Selection of sources of evidence†	9	State the process for selecting sources of evidence (i.e., screening and eligibility) included in the scoping review.	The Covidence platform was used to manage the review process. Papers were screened for relevance based on titles, abstracts, and full-text reviews. Six reviewers were involved, with disagreements resolved by discussion or a third reviewer.
Data charting process‡	10	Describe the methods of charting data from the included sources of evidence (e.g., calibrated forms or forms that have been tested by the team before their use, and whether data charting was done independently or in duplicate) and any processes for obtaining and confirming data from investigators.	Data was extracted using a standardized charting framework. Two reviewers independently charted the data, and disagreements were resolved collaboratively.
Data items	11	List and define all variables for which data were sought and any assumptions and simplifications made.	Extracted data included:<br>- Study characteristics (year, country, objectives).<br>- Biosecurity practices and principles.<br>- Farmer knowledge, attitudes, and perceptions (KAPs).<br>- Risk factors and recommendations for biosecurity improvement.
Critical appraisal of individual sources of evidence§	12	If done, provide a rationale for conducting a critical appraisal of included sources of evidence; describe the methods used and how this information was used in any data synthesis (if appropriate).	All papers chosen went through the steps of selection one at the title and abstract review step and were assessed based on a predetermined checklist in the covidence platform. Papers that passed title and abstract review went through full text review. This step was largely for data extraction but also served as a step to QC whether the paper was reliable. Each paper had to be vetted by atleast two research team members at all steps and any disgreements were resolved by a third reviewer.
Synthesis of results	13	Describe the methods of handling and summarizing the data that were charted.	Results were synthesized through thematic analysis, focusing on knowledge, attitudes, and practices (KAPs). Themes included barriers to adoption, critical biosecurity

SECTION	ITEM	PRISMA-ScR CHECKLIST ITEM	REPORTED ON PAGE #
			practices, and the role of technology in risk assessment.
**RESULTS**			
Selection of sources of evidence	14	Give numbers of sources of evidence screened, assessed for eligibility, and included in the review, with reasons for exclusions at each stage, ideally using a flow diagram.	A total of 431 records were identified (PubMed: 211; CAB Abstracts: 220). After deduplication and screening, 18 studies were included in the review. Reasons for exclusion included irrelevance to geographical scope, lack of focus on biosecurity, and non-original research.
Characteristics of sources of evidence	15	For each source of evidence, present characteristics for which data were charted and provide the citations.	The 18 studies predominantly originated from the USA (n=15) and Canada (n=3). Research focused on mixed swine farming systems, biosecurity practices, and risk assessments. Study designs included surveys, cross-sectional, and machine learning-based analyses.
Critical appraisal within sources of evidence	16	If done, present data on critical appraisal of included sources of evidence (see item 12).	The included studies varied in their focus, with some addressing disease-specific biosecurity risks and others exploring farmer attitudes and perceptions. Key gaps were identified, including limited representation from non-English studies.
Results of individual sources of evidence	17	For each included source of evidence, present the relevant data that were charted that relate to the review questions and objectives.	Each paper was reviewed for practices, attitudes and perceptions as well as knowledge on matters biosecurity in Nrth American swine farms. All 18 studies were extracted using a covidence data extraction template and a qualitative analysis of practices was also conducted and summarized in excel.
Synthesis of results	18	Summarize and/or present the charting results as they relate to the review questions and objectives.	Key findings include:<br>- Vehicle and personnel movement were the most frequently mentioned risks for disease transmission.<br>- Producer attitudes significantly influenced biosecurity adoption, with cost and perceived risk being major determinants.

SECTION	ITEM	PRISMA-ScR CHECKLIST ITEM	REPORTED ON PAGE #
			- Backyard and small-scale farms exhibited knowledge gaps and lax biosecurity measures.<br>- Critical biosecurity practices included quarantine protocols, defined biosecurity zones (PBA, LOS), and vehicle disinfection.
**DISCUSSION**			
Summary of evidence	19	Summarize the main results (including an overview of concepts, themes, and types of evidence available), link to the review questions and objectives, and consider the relevance to key groups.	The review highlights the importance of multi-layered biosecurity approaches, tailored education programs, and leveraging technology to enhance disease prevention. Variability in biosecurity adherence across farm types and regions underscores the need for flexible, scalable interventions.
Limitations	20	Discuss the limitations of the scoping review process.	Exclusion of non-English studies may have limited geographic and linguistic representation.<br>- Reliance on two databases (PubMed, CAB Abstracts) could have excluded relevant studies from other sources.
Conclusions	21	Provide a general interpretation of the results with respect to the review questions and objectives, as well as potential implications and/or next steps.	The findings emphasize the need for updated assessments, targeted interventions, and collaborative efforts to strengthen biosecurity in North American swine production systems. Effective biosecurity requires proactive engagement from all stakeholders and the integration of innovative technologies like machine learning.
**FUNDING**			
Funding	22	Describe sources of funding for the included sources of evidence, as well as sources of funding for the scoping review. Describe the role of the funders of the scoping review.	Funding for this project from USDA APHIS Award Number AP21VSSP0000C023. The funder had no other role or influence on how the study was conducted and the interpretations of the collected data.

JBI = Joanna Briggs Institute; PRISMA-ScR = Preferred Reporting Items for Systematic reviews and Meta-Analyses extension for Scoping Reviews.

* Where *sources of evidence* (see second footnote) are compiled from, such as bibliographic databases, social media platforms, and Web sites.
† A more inclusive/heterogeneous term used to account for the different types of evidence or data sources (e.g., quantitative and/or qualitative research, expert opinion, and policy documents) that may be eligible in a scoping review as opposed to only studies. This is not to be confused with *information sources* (see first footnote).
‡ The frameworks by Arksey and O'Malley (6) and Levac and colleagues (7) and the JBI guidance (4, 5) refer to the process of data extraction in a scoping review as data charting.
§ The process of systematically examining research evidence to assess its validity, results, and relevance before using it to inform a decision. This term is used for items 12 and 19 instead of "risk of bias" (which is more applicable to systematic reviews of interventions) to include and acknowledge the various sources of evidence that may be used in a scoping review (e.g., quantitative and/or qualitative research, expert opinion, and policy document).

From: Tricco AC, Lillie E, Zarin W, O'Brien KK, Colquhoun H, Levac D, et al. PRISMA Extension for Scoping Reviews (PRISMAScR): Checklist and Explanation. Ann Intern Med. 2018;169:467–473. doi: 10.7326/M18-0850.
