# Supplementary material for: A scoping review of knowledge, attitudes, and practices in swine farm biosecurity in North America
Source: Front Vet Sci. 2025 Mar 3;12:1507704. doi: 10.3389/fvets.2025.1507704 (PMC11911468; doi:10.3389/fvets.2025.1507704)
Supplement: Supplementary file 3 [file Data_Sheet_2.docx]

**Supplementary Data 2**

**Search Strategy for the Scoping Review on Biosecurity in Swine Farms in North America**

**Databases Searched**

The following electronic databases were searched to identify relevant literature:

1. **PubMed/MEDLINE**
2. **CAB Abstracts (via Ovid)**

**Search Strategy for PubMed**

We performed a key word search used in PubMed using the following search terms:

(Pigs OR swine) AND (farmer* OR producer) AND (biosecurity)

Key word search in PubMed searches all fields

**Filters Applied:**

- **Language:** English
- **Publication Date:** 2011–2022

**Date search performed:** July 12 2022

**Number of Results Retrieved:** 211

**Search Strategy for CAB Abstracts (via Ovid)**

Similar search terms were used in CAB Abstracts:

**Search Query:**

((pigs OR swine) AND (farmer* OR producer) AND (biosecurity)).af.

*(Note: .af. refers to "all fields" in CAB Abstracts.)*

**Filters Applied:**

- **Language:** English
- **Publication Date:** 2011–2022

**Date search performed:** July 12 2022

**Number of Results Retrieved:** 220

**Selection Process**

1. **Deduplication**: Records were first de-duplicated using EndNote, which removed 94 duplicate records.
2. **Screening in Covidence**: A total of 337 unique records were uploaded to covidence for screening. Fourteen (14) other duplicates were identified and removed by covidence.

**Inclusion and exclusion criteria were as listed in Table 1 of the manuscript:**

Inclusion criteria

- Focuses on swine production systems
- In English
- Study on Biosecurity and Disease Management in Swine Production systems
- Published between 2011 and 2022
- The study was carried out in North America
- Contains information on practices, production systems, the profile of producers, attitudes of producers towards biosecurity, or recommendations about biosecurity
- Original research papers

Exclusion criteria

- Non-swine production systems
- Not English
- No mention or focus on biosecurity or disease management
- Published before 2011.
- The study was carried out outside of North America
- Does not have information on practices, production systems, profile of producers, attitudes of producers towards biosecurity, or recommendations about biosecurity
- Non-research papers (ex, reviews, letters to the editor, case reports, etc.)

1. **Full-Text Review**: After title and abstract screening, 323 papers were retrieved for full-text review.
2. **Final Inclusion**: A total of **18 studies** were included in the scoping review.

A PRISMA flowchart summarizing the study selection process is provided in **Figure 1** of the manuscript.

**Rationale for Database Selection**

The selection of **PubMed/MEDLINE and CAB Abstracts** was based on their coverage of peer-reviewed biomedical and agricultural research, ensuring relevant literature was retrieved. While PubMed is widely used for medical and veterinary research, CAB Abstracts provides broader coverage of agricultural and animal health-related studies, including biosecurity-focused research.

Government documents and grey literature were only included to the extent of indexing in these databases. However, relevant government sources were incorporated into the discussion where applicable.

**Limitations of the Search Strategy**

- Studies published in languages other than English were excluded, which may have led to the omission of relevant research from non-English-speaking countries. While this restriction was due to the team’s constraints in resources, funding, and translation expertise, we note that both PubMed and CABI are multilingual databases.
- Grey literature was not systematically searched beyond what was indexed in the selected databases, potentially leading to gaps in government or industry reports.
- The use of two databases may have limited the retrieval of studies available in other sources such as Web of Science or Scopus.
